# Effect of Food on the Pediatric Dispersible Tablet Formulations of TRIUMEQ and DOVATO in Healthy Adult Participants

**DOI:** 10.3390/pharmaceutics15051470

**Published:** 2023-05-11

**Authors:** Hardik Chandasana, Ryan Marnoch, Michael McKenna, Julia Double, Ciara Seal, Gilda Bontempo, Allen Wolstenholme, Ann Buchanan

**Affiliations:** 1 Clinical Pharmacology Modeling and Simulation, GSK, Collegeville, PA 19426, USA; 2 Clinical Development, GSK, London TW8 9GS, UK; 3 Safety and Medical Governance, GSK, London TW8 9GS, UK; 4 In Vitro/In Vivo Translation, GSK, Collegeville, PA 19426, USA; 5 ViiV Healthcare, Branford, CT 06405, USA; 6 Clinical Development, GSK, Collegeville, PA 19426, USA; 7 USA ViiV Healthcare, Durham, NC 27701, USA

**Keywords:** food effect(s), HIV/AIDS, pharmacokinetics, pediatric, dispersible tablet, fixed-dose combination

## Abstract

This randomized food effect study in healthy adult participants examined dispersible tablet formulations of fixed-dose combinations of dolutegravir/abacavir/lamivudine (TRIUMEQ) and dolutegravir/lamivudine (DOVATO). While adult tablet formulations of these combinations are currently approved for the treatment of human immunodeficiency virus, alternate formulations for children are urgently needed to facilitate appropriate pediatric dosing for patients who may have difficulty swallowing a conventional tablet. This study compared the effect of a high-fat, high-calorie meal on the pharmacokinetics, safety, and tolerability of dispersible tablet (DT) formulations of the two-drug and three-drug regimens, with administration under fasting conditions. Both the two-drug and three-drug dispersible tablet formulations, administered under fasting conditions and following a high-fat, high-calorie meal, were well tolerated in healthy participants. There were no clinically relevant differences in drug exposure for either regimen when administered with a high-fat meal as compared to under fasting conditions. Safety observations were similar for both treatments, either in the fed or fasted state. Both TRIUMEQ DT and DOVATO DT formulations can be administer with or without food.

## 1. Introduction

In 2020, 36 million adults and 1.7 million children (aged 0 to 14 years) worldwide were living with human immunodeficiency virus (HIV) [1]. While 73% of adults had access to antiretroviral therapy (ART), only 54% of children had access to ART. It is also well known that viral suppression rates among children are starkly lower than those in adults. TRIUMEQ^®^ is a fixed-dose combination (FDC) that contains the integrase strand transfer inhibitor dolutegravir and two nucleoside reverse transcriptase inhibitors, abacavir and lamivudine. It was first approved for adults in the United States in 2014 [2]. In 2014, the adult FDC was approved in the European Union (EU) for adolescents 12 years and older weighing at least 40 kg, and in the US, it was approved for pediatric patients weighing 40 kg and above in 2017. The approved dose for the treatment of HIV infection in both adult and pediatric patients weighing at least 40 kg is 1 tablet (50 mg dolutegravir/600 mg abacavir/300 mg lamivudine), taken by mouth, administered once daily, with or without food [2].

DOVATO^®^ is an FDC two-drug regimen that contains dolutegravir (50 mg) and lamivudine (300 mg), and it was first approved in the US in 2019. In the EU, the dolutegravir and lamivudine FDC was approved in 2019 for adults and adolescents 12 years and older weighing at least 40 kg. The approved dose for the treatment of HIV infection in adults is 1 tablet by mouth (50 mg dolutegravir/300 mg abacavir), administered once daily, with or without food [3].

Alternate formulations (e.g., dispersible tablet [DT]) and dosing strategies for both the dolutegravir/lamivudine tablet and the dolutegravir/abacavir/lamivudine tablet have been developed for pediatric patients younger than 12 years old who may have difficulty swallowing conventional tablet formulations and to facilitate appropriate pediatric dosing. The development of a DT formulation of the two-drug and three-drug regimens would provide drug exposures similar to those of the approved dolutegravir/lamivudine tablet and dolutegravir/abacavir/lamivudine tablet formulations and be beneficial to patients weighing less than 25 kg who are unable to swallow tablet formulations (Table 1).

This study aimed to investigate the effect of a high-fat and high-calorie meal on the pharmacokinetics (PK), safety, and tolerability of the DT formulations of the dolutegravir/lamivudine tablet and the dolutegravir/abacavir/lamivudine tablet compared with administration under fasting conditions, in accordance with the US Food and Drug Administration (FDA) guidance regarding food effect bioavailability and fed bioequivalence studies [4].

## 2. Materials and Methods

### 2.1. Ethics and Good Clinical Practice

The study protocol, any amendments, the informed consent, and other information that required pre-approval were reviewed and approved by a national, regional, or investigational center ethics committee or institutional review board, in accordance with the International Council for Harmonization (ICH) Good Clinical Practice (GCP) and applicable country-specific requirements, including the US Title 21 Code of Federal Regulations (CFR) 312.3(b) for the constitution of independent ethics committees. The study was monitored in accordance with ICH E6, Section 5.18. Written informed consent was obtained from each participant prior to the performance of any study-specific procedures.

### 2.2. Study Design

This was a two-cohort, single-center, randomized, open-label, single-dose, crossover study in healthy volunteers. The study [Study 216149, (ClinicalTrials.gov Identifier NCT04827134)] was conducted in the US at PPD Development Inc. Clinics (Austin, TX, USA) and consisted of the following: a screening period (within 28 days before the first dose of study intervention); two treatment periods, with a single dose of study intervention per treatment period in each cohort; a washout period of 7 days; and a follow-up visit (7 to 14 days after the dose of the study intervention in Period 2). Cohort 1 (dolutegravir/abacavir/lamivudine DT treatments) enrolled 16 participants, and Cohort 2 (dolutegravir/lamivudine DT treatments) enrolled 17 participants. Enrolled healthy male and female participants were from 18 to 50 years of age and weighed more than or equal to 50 kg (male) or 45 kg (female). 

**Figure 1:**
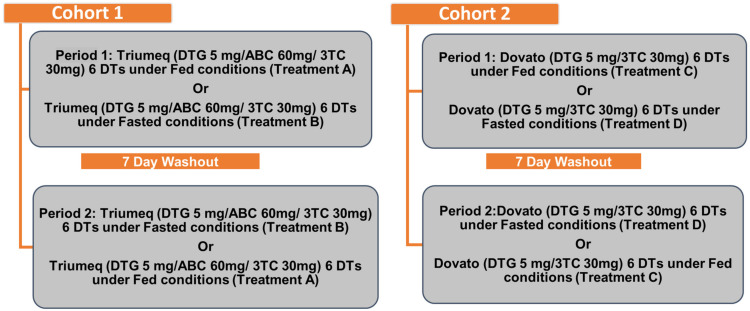
Study design.

In each cohort, prior to dosing on Day 1 of Period 1, the participants were randomly assigned to one of two treatment sequences (AB or BA in Cohort 1, and CD or DC in Cohort 2). Treatments A and B consisted of a single oral dose of dolutegravir 5 mg/abacavir 60 mg/lamivudine 30 mg × 6 DTs dispersed in 20 mL water (with a total water volume of 240 mL). Treatments C and D consisted of a single oral dose of dolutegravir 5 mg/lamivudine 30 mg × 6 DTs dispersed in 20 mL water (with a total water volume of 240 mL). Treatments A and C were administered under fed conditions. After an overnight fast for at least 10 h prior to dosing, participants received a high-fat and high-calorie meal (917 calories derived from 51.2% fat, 12.9% protein, and 36% carbohydrates) 30 min prior to dosing, and finishing the meal in 25 min or less. Treatments B and D were administered after an overnight fast of at least 10 h (Figure 1).

### 2.3. PK Assessments

PK samples were collected to evaluate dolutegravir, abacavir, and lamivudine plasma concentrations. Blood samples were acquired via an indwelling cannula (or by direct venipuncture), collected into a K2EDTA tube, and centrifuged for 10 min at approximately 1500× *g* within 60 min of collection. Supernatant plasma was transferred to an amber colored polypropylene sample storage tube and stored at −20 °C (±10 °C) before shipment. 

Plasma samples were analyzed for dolutegravir using a validated analytical method based on protein precipitation, followed by ultra performance liquid chromatography (UPLC) with tandem mass spectrometry (MS/MS) detection. The lower limit of quantification (LLQ) for dolutegravir was 20.0 ng/mL using a 25 μL aliquot of EDTA plasma. 

Plasma samples were analyzed for abacavir and lamivudine using a validated analytical method based on protein precipitation, followed by ultra high-performance liquid chromatography (UHPLC) with MS/MS detection. The LLQ was 2.50 ng/mL for abacavir and lamivudine, using a 50 μL aliquot of EDTA plasma. 

For each analytical method, quality control (QC) samples containing either dolutegravir, at six different concentrations, or abacavir and lamivudine, at five different concentrations, were analyzed with each batch of samples against separately prepared calibration standards. For the analysis to be acceptable, no more than one-third of the QC results could deviate from the nominal concentration by more than 15%, and at least 50% of the results from each QC concentration should be within 15% of nominal.

Plasma dolutegravir, abacavir, and lamivudine concentration–time data were analyzed using noncompartmental methods with Phoenix WinNonlin version 8.0. Calculations were based on the actual sampling times recorded during the study. The statistical analysis using analysis of variance (ANOVA) regarding the effect of food (high-fat, high-calorie meal) was presented in a tabular format with geometric mean ratios for the following treatment comparisons: Treatment A (fed) versus Treatment B (fasted), and Treatment C (fed) versus Treatment D (fasted).

Analyses were performed on the natural logarithms of the area under the concentration–time curve (AUC) extrapolated to infinity [AUC(0-inf)], AUC from time 0 to time of the last observed quantifiable concentration [AUC(0-t)], and maximum observed plasma concentration (Cmax) using linear mixed effect models with the treatment, sequence, and period as fixed effects and the participant nested within the sequence [participant(sequence)] as a random effect. Point estimates and 90% confidence intervals (CI), [estimate ± SE × tinv(0.95, df)], and the intra-participant coefficient of variation (CV%) [sqrt (exp (variance for ln-transformed data)-) × 100] for treatment differences on the log scale derived from the model were exponentiated to obtain estimates for geometric mean ratios and CIs on the original scale.

### 2.4. Safety Assessments

Safety assessments included the monitoring of adverse events (AEs), clinical laboratory tests, vital signs, electrocardiograms (ECGs), physical examinations, pregnancy tests (for female participants), toxicity events, and COVID-19 infections. AE information volunteered by the participant, discovered by investigator questioning, or detected by other means was collected from the start of the study treatment until the follow-up contact. The following information on AEs was obtained: duration (start and stop dates), severity (mild, moderate, severe), causality (reasonable possibility, yes/no), actions taken, and outcome.

### 2.5. Palatability Assessments

A palatability questionnaire was provided to each participant following the dosing of dispersion treatments. Participants were given the questionnaire to read prior to receiving each unique dispersion dose. The palatability questionnaire variables were summarized descriptively.

## 3. Results

### 3.1. Participant Disposition and Demographics

Healthy male and female participants were enrolled into Cohort 1 (*n* = 16, dolutegravir/abacavir/lamivudine DT treatments) and Cohort 2 (*n* = 17, dolutegravir/lamivudine DT treatments). The participants in both Cohort 1 and 2 were predominantly male (63% and 76%). Body mass index, height, weight, ethnicity, and race were comparable between the two cohorts (Table 2).

### 3.2. Cohort 1 Plasma PK Parameters of Dolutegravir, Abacavir, and Lamivudine

When a single dose of the dolutegravir/abacavir/lamivudine DT was administered under fed conditions, the dolutegravir geometric mean Cmax was approximately 29% lower than when administered under fasted conditions (Table 3 and Table 4). The time to maximum observed concentration (Tmax) values were delayed under fed conditions compared with fasted conditions, with median Tmax values of 5.00 h and 1.25 h, respectively (Table 3). The geometric mean AUC(0-inf), AUC(0-t), and AUC from time 0 to 24 h [AUC(0-24)] values were 12%, 13%, and 17% lower under fed conditions, respectively, compared with fasted conditions. The dolutegravir geometric mean concentration at 24 h post-dose (C24) and the last quantifiable concentration (Ct) values were similar for fed and fasted conditions. The between-participant percent coefficient of variation (%CVb) values were generally moderate across all parameters and treatments, with the highest variability observed for Ct.

When a single dose of the dolutegravir/abacavir/lamivudine DT was administered under fed conditions, the abacavir geometric mean Cmax values were approximately 55% lower than when administered under fasted conditions (Table 3 and Table 4). The Tmax values were delayed under fed conditions compared with fasted conditions, with median Tmax values of 2.75 h and 0.50 h (Table 3). The abacavir geometric mean AUC(0-inf), AUC(0-t), and AUC(0-24) values were each 14% lower under fed conditions compared with fasted conditions. The abacavir geometric mean C24 and Ct values were similar for fed and fasted conditions. The %CVb values were generally moderate across parameters and treatments, with the highest variability observed for Ct.

When a single dose of the dolutegravir/abacavir/lamivudine DTs was administered under fed conditions, the lamivudine geometric mean Cmax values were approximately 36% lower than when administered under fasted conditions (Table 3 and Table 4). The Tmax values were delayed under fed conditions compared with fasted conditions, with median Tmax values of 3.50 h and 1.50 h (Table 3). The lamivudine geometric mean AUC(0-inf), AUC(0-t), and AUC(0-24) values were 11%, 12%, and 13% lower, respectively, under fed conditions compared with fasted conditions. The lamivudine geometric mean C24 and Ct values were similar for fed and fasted conditions. The %CVb values were moderate across parameters and treatments.

**Figure 2:**
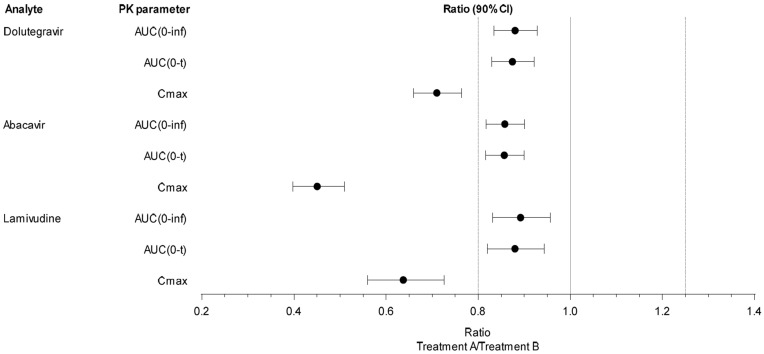
Ratios of GLS means and 90% CIs (TRIUMEQ DT Fed (Treatment A) vs. TRIUMEQ DT Fasted (Treatment B)) for primary PK parameters. Treatment A = TRIUMEQ (dolutegravir 5 mg/abacavir 60 mg/lamivudine 30 mg) 6 DTs dispersed in 20 mL water (fed); Treatment B = TRIUMEQ (dolutegravir 5 mg/abacavir 60 mg/lamivudine 30 mg) 6 DTs dispersed in 20 mL water (fasted); CI = confidence interval.

When a single dose of the dolutegravir/abacavir/lamivudine DT was administered under fed conditions, mean peak plasma exposures (Cmax) for dolutegravir, abacavir, and lamivudine were lower than under fasted conditions, with geometric least squares (LS) means ratios of 0.7102, 0.4503, and 0.6373, respectively (Table 4, Figure 2). 

Under fed conditions, mean total plasma exposures [AUC(0-inf)] for dolutegravir, abacavir, and lamivudine were slightly lower than under fasted conditions, with geometric LS means ratios of 0.8800, 0.8578, and 0.8919, respectively (Table 4, Figure 2). 

Under fed conditions, the mean total plasma exposures [AUC(0-t)] for dolutegravir, abacavir, and lamivudine were slightly lower than under fasted conditions, with geometric LS means ratios of 0.8744, 0.8567, and 0.8798, respectively (Table 4, Figure 2). 

### 3.3. Cohort 2 Plasma PK Parameters of Dolutegravir and Lamivudine

When a single dose of the dolutegravir/lamivudine DT was administered under fed conditions, the dolutegravir geometric mean Cmax values were approximately 27% lower than when administered under fasted conditions (Table 5 and Table 6). The Tmax values were delayed under fed conditions compared with fasted conditions, with median Tmax values of 4.00 h and 0.875 h (Table 5). The geometric mean AUC(0-inf), AUC(0-t), and AUC(0-24) values were similar compared with those under fasted conditions. The dolutegravir geometric mean C24 and Ct values were similar for fed and fasted conditions. The %CVb values were generally moderate across parameters and treatments, with the highest variability observed for Ct.

When a single dose of the dolutegravir/lamivudine DT was administered under fed conditions, the lamivudine geometric mean Cmax values were approximately 48% lower than those noted under fasted conditions (Table 5 and Table 6). The Tmax values were delayed under fed conditions compared with those under fasted conditions, with median Tmax values of 3.00 h and 0.750 h (Table 5). The lamivudine geometric mean AUC(0-inf), AUC(0-t), and AUC(0-24) values were similar under fed conditions compared with fasted conditions. The lamivudine geometric mean C24 and Ct values were similar for fed and fasted conditions. The %CVb values were moderate across parameters and treatments.

**Figure 3:**
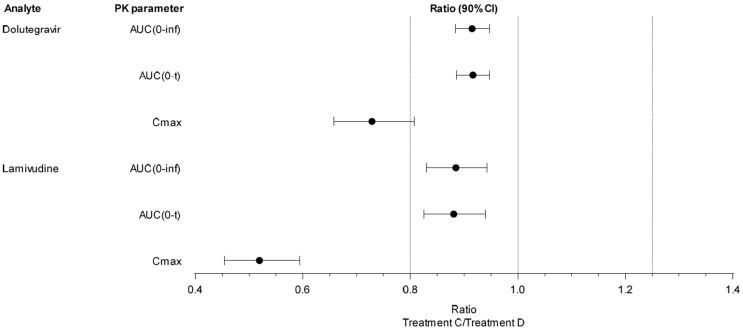
Ratios of GLS means and 90% CIs (DOVATO DT Fed (Treatment C) vs. DOVATO DT Fasted (Treatment D)) for primary PK parameters. Treatment C = DOVATO (dolutegravir 5 mg/lamivudine 30 mg) 6 DTs dispersed in 20 mL water (fed); Treatment D = DOVATO (dolutegravir 5 mg/lamivudine 30 mg) 6 DTs dispersed in 20 mL water (fasted); CI = confidence interval.

When a single dose of the dolutegravir/lamivudine DT was administered under fed conditions, mean peak plasma exposures (Cmax) for dolutegravir and lamivudine were lower than those under fasted conditions, with geometric LS means ratios of 0.7284 and 0.5191, respectively (Table 6, Figure 3). 

Under fed conditions, mean total plasma exposures [AUC(0-inf)] for dolutegravir and lamivudine were slightly lower than those under fasted conditions, with geometric LS means ratios of 0.9148 and 0.8847, respectively (Table 6, Figure 3). 

Under fed conditions, mean total plasma exposures [AUC(0-t)] for dolutegravir and lamivudine were slightly lower than those under fasted conditions, with geometric LS means ratios of 0.9163 and 0.8806, respectively (Table 6, Figure 3). 

### 3.4. Safety Results

No deaths, serious AEs, or withdrawals due to AEs were reported during the study in either Cohort 1 or Cohort 2. All AEs were mild (Grade 1) in intensity and were resolved. Additionally, no clinical laboratory evaluations, liver events, ECGs, or vital sign changes were reported as AEs in either Cohort 1 or Cohort 2.

### 3.5. Cohort 1

There were five AEs reported across four participants (25%) in Cohort 1 of the study (Table 7). One participant (6%) had abdominal discomfort after receiving dolutegravir/abacavir/lamivudine 6 DTs dispersion under fed conditions (Treatment A). Three participants (19%) experienced AEs, with a total of four AEs reported (angular cheilitis, oropharyngeal pain, nausea, and headache), after receiving dolutegravir/abacavir/lamivudine 6 DTs dispersion under fasting conditions (Treatment B). A total of two drug-related AEs (headache and nausea) were reported in one participant after receiving Treatment B. All AEs were mild (Grade 1) in intensity and resolved within a week following discharge from the unit. One participant (6%) in Cohort 1 had two AEs (headache and nausea) that were considered drug-related by the investigator. Both events occurred approximately 4.5 h after receiving Treatment B in Period 1 and resolved approximately 3.5 h after onset, without additional treatment.

### 3.6. Cohort 2

A total of two mild (Grade 1) AEs (dry skin in Period 1 [Treatment D] and skin irritation in Period 2 [Treatment C]) were reported in one participant in Cohort 2 of the study (Table 7). Both events resolved within 4 days of the onset of AEs. No drug-related AEs were reported in Cohort 2 of the study.

### 3.7. Palatability Results

Following administration of dolutegravir/abacavir/lamivudine DTs as a dispersion (Cohort 1), 2 participants (13%) rated the palatability of the dispersion as very good, 14 participants (88%) rated the palatability as neutral/acceptable, and 5 participants (31%) rated the palatability as very poor in one or both treatment periods (Table 8). No difference was noted in response to the palatability questionnaire for Treatment A (fed) versus Treatment B (fasted).

Following administration of dolutegravir/lamivudine DT as a dispersion (Cohort 2), 13 participants (76%) rated the palatability of the dispersion as very good, and 10 participants (59%) rated the palatability as neutral/acceptable in one or both the treatment periods (Table 8). No difference was noted in response to the palatability questionnaire for Treatment C (fed) versus Treatment D (fasted).

## 4. Discussion

### 4.1. Pharmacokinetics

#### 4.1.1. Dolutegravir

Dolutegravir Cmax was reduced by approximately 27% (dolutegravir/lamivudine DT) to 29% (dolutegravir/abacavir/lamivudine DT) following administration with a high-fat meal versus while fasting, while dolutegravir AUC and C24 were unaffected by food status. The decrease in the Cmax coupled with a delay in Tmax was likely a consequence of delayed gastric emptying with meal coadministration. The decrease in the rate, but not the extent, of dolutegravir absorption with a high-fat meal is not considered clinically significant. 

In a previous food effect study with dolutegravir/abacavir/lamivudine tablets, dolutegravir Cmax and AUC were increased by 37% and 48%, respectively, with a high-fat meal as compared to a fasted state [5]. Similarly, dolutegravir Cmax and AUC were increased by 21% and 33%, respectively, with a high-fat meal as compared to the fasted state when dolutegravir/lamivudine tablets were administered with a high-fat meal [6]. These increases in dolutegravir exposures with the FDC formulations when administered with food were comparable to the previously observed increase in dolutegravir exposure (66–67%) when TIVICAY (dolutegravir) tablets were administered in the presence of food [7]. Dolutegravir is a biopharmaceutical classification system (BCS) class 2 compound, and the increase in exposure when these solid tablet formulations were administered in the presence of a high-fat meal is probably due to an increase in overall solubility. Based on the accumulated safety data in Phase 2b and Phase 3 studies, the increase in dolutegravir exposure with a high-fat meal was not considered to be clinically significant, which permitted dolutegravir dosing without restrictions regarding food or food content [2,8].

Unlike the results of previous studies of the tablet formulation, dolutegravir AUC and C24 were not impacted when dolutegravir/abacavir/lamivudine DT or dolutegravir/lamivudine DT were administered as a dispersion in the presence of a high-fat meal. In the fasted state, the dolutegravir/abacavir/lamivudine DT and dolutegravir/lamivudine DT dispersion showed higher dolutegravir bioavailability (~60% to 70%) compared to the corresponding whole tablets, suggesting that there is no further room to improve solubility in the presence of a high-fat meal [9].

#### 4.1.2. Abacavir

There was an approximate 55% reduction in the abacavir Cmax and no change in the abacavir AUC when dolutegravir/abacavir/lamivudine DTs were administered in the presence of a high-fat meal versus in a fasted state. The decrease in the abacavir Cmax coupled with a delay in Tmax was likely a consequence of delayed gastric emptying with meal coadministration. In prior studies, minor decreases in abacavir Cmax were observed, with no changes in AUC when abacavir was administered as the FDC formulations [5,10] or as a single agent [11,12] with a high-fat meal as compared to in a fasted state. Based on the observed lack of a clinically relevant food effect on abacavir exposures across combinations and formulations, the decrease in the Cmax with dolutegravir/abacavir/lamivudine DT is not considered to be clinically relevant.

#### 4.1.3. Lamivudine

There was an approximate 36% and 48% reduction in lamivudine Cmax and no change in lamivudine AUC when administered as dolutegravir/abacavir/lamivudine DTs and dolutegravir/lamivudine DTs, respectively, in presence of a high-fat meal versus in a fasted state. The decrease in the lamivudine Cmax coupled with a delay in Tmax was likely a consequence of delayed gastric emptying with meal coadministration. This result is similar to that of previous studies [6], in which the lamivudine Cmax was approximately 40% and 32% lower when administered with a standard high-fat meal, without any change in AUC. In the other historical food effect studies, there were no clinically relevant changes in lamivudine AUC or Cmax when administered while fasting or with a high-fat meal [6,10,13]. Based on the observed lack of a clinically relevant food effect on lamivudine exposures across combinations and formulations, the decrease in the Cmax with dolutegravir/abacavir/lamivudine DT is not considered to be clinically relevant.

### 4.2. Safety and Tolerability

Both dolutegravir/abacavir/lamivudine DT and dolutegravir/lamivudine DT were well tolerated, and no new safety signals were observed. Safety data were similar across both the treatments (fed versus fasted) for dolutegravir/abacavir/lamivudine DT and dolutegravir/lamivudine DT. No deaths, serious AEs, or withdrawals due to AEs were reported for either cohort. Additionally, no clinically significant trends in clinical laboratory values, vital signs, or ECGs were reported as AEs.

### 4.3. Palatability

The palatability of dolutegravir/abacavir/lamivudine DT and dolutegravir/lamivudine DT administered as a dispersion was rated neutral/acceptable or better by most (Cohort 1) or all (Cohort 2) of the participants in both the treatment periods.

## 5. Conclusions

While the Cmax of the constituent compounds was reduced following administration of the TRIUMEQ DT and the DOVATO DT formulations in the context of a high-fat meal, these reductions were not clinically relevant regarding the pharmacokinetic parameters important for efficacy, C24 (dolutegravir) and AUC (abacavir [Cohort 1 only] and lamivudine). No clinically significant trends in clinical laboratory values, vital signs, or ECGs were observed or reported as AEs. Safety data were similar across both the treatments (fed vs. fasted) and for both formulations. Therefore, both TRIUMEQ DT and DOVATO DT may be administered with or without food, and these results provide useful information regarding formulation administration to facilitate appropriate pediatric dosing. 

## Figures and Tables

**Table 1:** Fixed dose formulations.

Product Name	Drugs and Doses
TRIUMEQ FDC	50 mg Dolutegravir/600 mg Abacavir/300 mg Lamivudine
TRIUMEQ DT FDC	5 mg Dolutegravir/60 mg Abacavir/30 mg Lamivudine
DOVATO FDC	50 mg Dolutegravir/300 mg Lamivudine
DOVATO DT FDC	5 mg Dolutegravir/30 mg Lamivudine

**Table 2:** Participant demographics.

Demographics	TRIUMEQ DT (Cohort 1) (N = 16)	DOVATO DT (Cohort 2) (N = 17)
Age in Years [Mean (SD)]	34.9 (8.10)	36.0 (8.07)
Sex [*n* (%)]		
Female, potentially able to bear children	6 (38)	4 (24)
Male	10 (63)	13 (76)
BMI (kg/m^2^) [Mean (SD)]	27.68 (1.909)	26.78 (2.520)
Height (cm) [Mean (SD)]	169.91 (12.780)	172.57 (8.070)
Weight (kg) [Mean (SD)]	80.21 (12.392)	80.13 (11.760)
Ethnicity [*n* (%)]		
Hispanic or Latino	9 (56)	7 (41)
Not Hispanic or Latino	7 (44)	10 (59)
Race [*n* (%)]		
White—White/Caucasian/European Heritage	9 (56)	9 (53)
Black or African American	5 (31)	6 (35)
White—Arabic/North African Heritage	1 (6)	1 (6)
Multiple, Black or African American and White	1 (6)	0
Asian—Central/South Asian Heritage	0	1 (6)

BMI = body mass index; SD = standard deviation. Note: For the purpose of calculating age, the ‘30th June’ has been imputed as the day and month of birth for all participants, as only year of birth was recorded. Cohort 1 included Treatments A and B, and Cohort 2 included Treatments C and D. Treatment A = TRIUMEQ (dolutegravir 5 mg/abacavir 60 mg/lamivudine 30 mg) 6 DTs dispersed in 20 mL water (fed); Treatment B = TRIUMEQ (dolutegravir 5 mg/abacavir 60 mg/lamivudine 30 mg) 6 DTs dispersed in 20 mL water (fasted); Treatment C = DOVATO (dolutegravir 5 mg/lamivudine 30 mg) 6 DTs dispersed in 20 mL water (fed); Treatment D = DOVATO (dolutegravir 5 mg/lamivudine 30 mg) 6 DTs dispersed in 20 mL water (fasted).

**Table 3:** Summary statistics of dolutegravir, abacavir, and lamivudine PK parameters—TRIUMEQ DT (Cohort 1).

	Analyte: Dolutegravir		Analyte: Abacavir		Analyte: Lamivudine	
Parameter	Treatment A (N = 16)	Treatment B (N = 16)	Treatment A (N = 16)	Treatment B (N = 16)	Treatment A (N = 16)	Treatment B (N = 16)
**Cmax (ng/mL)**						
Geometric Mean	2359	3322	1837	4080	886.9	1392
%CVb	17.6	20.6	21.1	32.3	18.9	33.3
**AUC(0-inf) (h × ng/mL) ^a^**						
Geometric Mean	59,140	67,210	8565	9986	6508	7295
%CVb	25.7	24.0	24.9	28.8	19.9	23.4
**AUC(0-t) (h × ng/mL)**						
Geometric Mean	55,770	63,790	8501	9923	6319	7182
%CVb	23.8	22.7	25.4	28.9	18.9	23.5
**AUC(0-24) (h × ng/mL)**						
Geometric Mean	36,380	43870	8553	9980	5817	6707
%CVb	19.4	20.1	24.8	28.8	18.1	24.4
**Tmax (h)**						
Median	5.000	1.250	2.750	0.500	3.500	1.508
(Min, Max)	(3.00, 12.00)	(0.50, 4.00)	(0.50, 4.00)	(0.25, 1.50)	(2.00, 4.00)	(0.50, 3.50)
**C24 (ng/mL) ^b^**						
Geometric Mean	958.6	996.3	4.833	3.814	23.40	21.74
%CVb	28.3	26.3	52.3	27.2	24.4	23.1

CI = confidence interval; %CVb = between participant percent coefficient of variation; **^a^** Treatment A lamivudine *n* = 15; **^b^** Treatment A abacavir *n* = 10, Treatment B abacavir *n* = 4; Treatment A = TRIUMEQ (dolutegravir 5 mg/abacavir 60 mg/lamivudine 30 mg) 6 DTs dispersed in 20 mL water (fed); Treatment B = TRIUMEQ (dolutegravir 5 mg/abacavir 60 mg/lamivudine 30 mg) 6 DTs dispersed in 20 mL water (fasted).

**Table 4:** Statistical analysis of dolutegravir, abacavir, and lamivudine PK parameters –TRIUMEQ DT (Cohort 1).

Parameter	Treatment	N	*n*	Geometric LS Means	Ratio of Geometric LS Means (A/B)	90% CI of the Ratio	Intra-Subject CV
**Analyte: Dolutegravir**							
Cmax (ng/mL)	A	16	16	2359	0.7102	(0.6598, 0.7643)	11.8
	B	16	16	3322			
AUC(0-inf) (h × ng/mL)	A	16	16	59,140	0.8800	(0.8344, 0.9282)	8.6
	B	16	16	67,210			
AUC(0-t) (h × ng/mL)	A	16	16	55,770	0.8744	(0.8293, 0.9219)	8.5
	B	16	16	63,790			
**Analyte: Abacavir**							
Cmax (ng/mL)	A	16	16	1837	0.4503	(0.3976, 0.5100)	20.2
	B	16	16	4080			
AUC(0-inf) (h × ng/mL)	A	16	16	8565	0.8578	(0.8168, 0.9007)	7.9
	B	16	16	9986			
AUC(0-t) (h × ng/mL)	A	16	16	8501	0.8567	(0.8159, 0.8995)	7.9
	B	16	16	9923			
**Analyte: Lamivudine**							
Cmax (ng/mL)	A	16	16	886.9	0.6373	(0.5594, 0.7260)	21.2
	B	16	16	1392			
AUC(0-inf) (h × ng/mL)	A	16	15	6511	0.8919	(0.8316, 0.9566)	10.9
	B	16	16	7300			
AUC(0-t) (h × ng/mL)	A	16	16	6319	0.8798	(0.8202, 0.9438)	11.3
	B	16	16	7182			

CI = confidence interval; CV = coefficient of variation; LS = least squares; Note: An analysis of variance (ANOVA) including treatment, period, and sequence as fixed effects and the participant nested within a sequence as a random effect was performed on the natural ln-transformed parameters AUC_(0-inf)_, AUC_(0-t)_, and C_max_. Treatment A = TRIUMEQ (dolutegravir 5 mg/abacavir 60 mg/lamivudine 30 mg) 6 DTs dispersed in 20 mL water (fed); Treatment B = TRIUMEQ (dolutegravir 5 mg/abacavir 60 mg/lamivudine 30 mg) 6 DTs dispersed in 20 mL water (fasted).

**Table 5:** Summary statistics of dolutegravir and lamivudine PK parameters—DOVATO DT (Cohort 2).

	Analyte: Dolutegravir		Analyte: Lamivudine	
Parameter	Treatment C (N = 17)	Treatment D (N = 16)	Treatment C (N = 17)	Treatment D (N = 16)
**Cmax (ng/mL)**				
Geometric Mean	2331	3131	1084	2066
%CVb	15.4	19.8	18.6	28.6
**AUC(0-inf) (h × ng/mL) ^a^**				
Geometric Mean	47,830	51,800	6975	7864
%CVb	24.2	24.3	10.1	13.9
**AUC(0-t) (h × ng/mL)**				
Geometric Mean	46,060	49,690	6799	7762
%CVb	22.2	22.0	10.5	14.0
**AUC(0-24) (h × ng/mL)**				
Geometric Mean	32,620	35,360	6339	7339
%CVb	19.2	15.6	10.7	14.9
**Tmax (h)**				
Median	4.000	0.875	3.000	0.750
(Min, Max)	(3.50, 8.00)	(0.50, 3.50)	(0.75, 4.00)	(0.50, 1.00)
**C24 (ng/mL)**				
Geometric Mean	764.7	715.4	22.56	20.38
%CVb	28.5	27.7	18.8	18.6

CI = confidence interval; %CVb = between participant percent coefficient of variation; **^a^** Treatment C *n* = 16; Treatment C = DOVATO (dolutegravir 5 mg/lamivudine 30 mg) 6 DTs dispersed in 20 mL water (fed); Treatment D = DOVATO (dolutegravir 5 mg/lamivudine 30 mg) 6 DTs dispersed in 20 mL water (fasted).

**Table 6:** Statistical analysis of dolutegravir and lamivudine PK parameters—DOVATO DT (Cohort 2).

Parameter	Treatment	N	*n*	Geometric LS Means	Ratio of Geometric LS Means (C/D)	90% CI of the Ratio	Intra-Subject CV
**Analyte: Dolutegravir**							
Cmax (ng/mL)	C	17	17	2306	0.7284	(0.6576, 0.8068)	16.4
	D	16	16	3166			
AUC(0-inf) (h × ng/mL)	C	17	16	47,610	0.9148	(0.8834, 0.9472)	5.6
	D	16	16	52,040			
AUC(0-t) (h × ng/mL)	C	17	17	45,800	0.9163	(0.8862, 0.9475)	5.3
	D	16	16	49,980			
**Analyte: Lamivudine**							
Cmax (ng/mL)	C	17	17	1079	0.5191	(0.4535, 0.5943)	21.8
	D	16	16	2078			
AUC(0-inf) (h × ng/mL)	C	17	16	6966	0.8847	(0.8303, 0.9426)	10.1
	D	16	16	7874			
AUC(0-t) (h × ng/mL)	C	17	17	6816	0.8806	(0.8251, 0.9398)	10.4
	D	16	16	7741			

CI = confidence interval; CV = coefficient of variation; LS = least squares. Note: An analysis of variance (ANOVA) including treatment, period, and sequence as fixed effects and the participant nested within a sequence as a random effect was performed on the natural ln-transformed parameters AUC_(0-inf)_, AUC_(0-t)_, and C_max_. Treatment C = DOVATO (dolutegravir 5 mg/lamivudine 30 mg) 6 DTs dispersed in 20 mL water (fed); Treatment D = DOVATO (dolutegravir 5 mg/lamivudine 30 mg) 6 DTs dispersed in 20 mL water (fasted).

**Table 7:** Summary of AEs by SOC and preferred term—TRIUMEQ DT (Cohort 1) and DOVATO DT (Cohort 2).

Treatment: TRIUMEQ DT			
SOC Preferred Term, *n* (%) [Number of AEs Reported]	Treatment A (N = 16)	Treatment B (N = 16)	Total (N = 16)
Any AE	1 (6) [1]	3 (19) [4]	4 (25) [5]
**Gastrointestinal disorders**, any AE	1 (6) [1]	2 (13) [2]	3 (19) [3]
Abdominal discomfort	1 (6) [1]	0	1 (6) [1]
Angular cheilitis	0	1 (6) [1]	1 (6) [1]
Nausea	0	1 (6) [1]	1 (6) [1]
**Nervous system disorders**, any AE	0	1 (6) [1]	1 (6) [1]
Headache	0	1 (6) [1]	1 (6) [1]
**Respiratory, thoracic, and mediastinal disorders**, any AE	0	1 (6) [1]	1 (6) [1]
Oropharyngeal pain	0	1 (6) [1]	1 (6) [1]
**Treatment: DOVATO DT**			
**SOC Preferred Term,** ***n* (%) [number of AEs reported]**	**Treatment C** **(N = 17)**	**Treatment D (N = 16)**	**Total (N = 17)**
Any AE	1 (6) [1]	1 (6) [1]	1 * (6) [2]
**Skin and subcutaneous tissue disorders**, any AE	1 (6) [1]	1 (6) [1]	1 (6) [2]
Dry skin	0	1 (6) [1]	1 (6) [1]
Skin irritation	1 (6) [1]	0	1 (6) [1]

AE = adverse event; SOC = system organ class. * This represents one subject with 2 AEs; Note: At each level of participant summarization, a participant is counted once if the participant reported one or more events. Treatment A = TRIUMEQ (dolutegravir 5 mg/abacavir 60 mg/lamivudine 30 mg) 6 DTs dispersed in 20 mL water (fed); Treatment B = TRIUMEQ (dolutegravir 5 mg/abacavir 60 mg/lamivudine 30 mg) 6 DTs dispersed in 20 mL water (fasted); Treatment C = DOVATO (dolutegravir 5 mg/lamivudine 30 mg) 6 DTs dispersed in 20 mL water (fed); Treatment D = DOVATO (dolutegravir 5 mg/lamivudine 30 mg) 6 DTs dispersed in 20 mL water (fasted).

**Table 8:** Summary of palatability of TRIUMEQ DT and DOVATO DT.

	TRIUMEQ DT (Cohort 1)		DOVATO DT (Cohort 2)	
Palatability	Treatment A (N = 16)	Treatment B (N = 16)	Treatment C (N = 17)	Treatment D (N = 16)
1-Very Poor	3 (19%)	4 (25%)	0	0
2-Neutral/Acceptable	13 (81%)	10 (63%)	8 (47%)	6 (38%)
3-Very Good	0	2 (13%)	9 (53%)	10 (63%)

Palatability: 1 = Very Poor, 2 = Neutral/Acceptable, 3 = Very Good; Treatment A = dolutegravir 5 mg/abacavir 60 mg/lamivudine 30 mg 6 DTs dispersed in 20 mL water (fed); Treatment B = dolutegravir 5 mg/abacavir 60 mg/lamivudine 30 mg 6 DTs dispersed in 20 mL water (fasted); Treatment C = dolutegravir 5 mg/lamivudine 30 mg 6 DTs dispersed in 20 mL water (fed); Treatment D = dolutegravir 5 mg/lamivudine 30 mg 6 DTs dispersed in 20 mL water (fasted).

## Data Availability

The data presented in this study are available on request from the corresponding author. The data are not publicly available due to privacy issues.
